# Enhancing Survival Outcome Predictions in Metastatic Non-Small Cell Lung Cancer Through PET Radiomics Analysis

**DOI:** 10.3390/cancers16223731

**Published:** 2024-11-05

**Authors:** Shuo Wang, Darryl Belemlilga, Yu Lei, Apar Kishor P Ganti, Chi Lin, Samia Asif, Jacob T Marasco, Kyuhak Oh, Sumin Zhou

**Affiliations:** 1 Department of Radiation Oncology, University of Nebraska Medical Center, Omaha, NE 68198, USA; dbelemlilga@unomaha.edu (D.B.); yu.lei@unmc.edu (Y.L.); clin@unmc.edu (C.L.); jmarasco@unmc.edu (J.T.M.); kyoh@unmc.edu (K.O.); szhou@unmc.edu (S.Z.); 2 College of Arts and Sciences, University of Nebraska Omaha, Omaha, NE 68182, USA; 3 Division of Oncology and Hematology, Department of Internal Medicine, VA Nebraska Western Iowa Health Care System, Omaha, NE 68105, USA; aganti@unmc.edu; 4 Division of Oncology and Hematology, Department of Internal Medicine, University of Nebraska Medical Center, Omaha, NE 68105, USA; samia.asif@unmc.edu

**Keywords:** radiomics, positron emission tomography, non-small cell lung cancer (NSCLC)

## Abstract

(1) Background: Advanced-stage lung cancer poses significant management challenges. The goal of this study was to identify crucial clinical and PET radiomics features that enable prognostic stratification for predicting outcomes. (2) Methods: PET radiomics features of the primary lung lesions were extracted from 99 patients with stage IVB NSCLC, and the robustness of these PET radiomics features was evaluated against uncertainties stemming from extraction parameters and contour variation. We trained three survival risk models (clinical, radiomics, and a composite) through a penalized Cox model framework. We also created a Balanced Random Forest classification predictive model, using the selected features, to predict 1-year survival. (3) Results: We identified 367 common PET radiomics features that exhibited robustness to perturbations introduced by contour variation and extraction parameters. Our findings indicated that both the radiomics and the composite model outperformed the clinical model in stratifying the risk for survival with statistical significance. In predicting 1-year survival, the radiomics model and the composite model also achieved better predicting accuracies compared to the clinical model. (4) Conclusions: Robust PET radiomics analysis successfully facilitated the stratification of patient risk for survival outcomes and predicted 1-year survival in stage IVB NSCLC.

## Simple Summary

It is important for patients to understand their chances of survival when they are diagnosed with advanced-stage lung cancer. A Positron Emission Tomography (PET) scan is an important type of imaging that shows how cancer is affecting the body’s metabolism and energy use. Radiomics is an exciting and growing field of research that looks for hidden patterns in medical images, like PET scans, to find potential signs that can help predict clinical outcomes such as a patient’s chances of survival. In our study, we identified robust radiomics features from PET scans that can accurately predict a patient’s survival likelihood, and these predictions are more accurate than using traditional clinical features alone. Our study could potentially help both doctors and patients better understand the chances of survival for those with advanced-stage lung cancer in the future.

## 1. Introduction

In the United States, lung cancer continues to be the leading cause of cancer deaths, representing over 20% of all cancer-related mortality [1]. Although lung cancer is being detected at progressively earlier stages with the advent of improved screening technologies, most of the lung cancer patients are diagnosed at an advanced stage [2]. Non-small cell lung cancer (NSCLC) is the predominant type, accounting for more than 80% of all lung cancers [3]. Overall cure and survival rates for advanced-stage NSCLC remain low due to the heterogeneity of the disease, resistance to traditional chemotherapies, and the limited effectiveness of targeted therapies and immunotherapies [4,5,6]. Therefore, prognostic stratification in advanced-stage NSCLC is a challenging yet important task due to the disease’s complex biology and the variability in treatment response.

Positron Emission Tomography (PET) imaging plays a crucial role in the diagnosis and management of lung cancer since it detects metabolically active tissues, such as malignant tumors, using radiotracers such as fluorodeoxyglucose (FDG) [7]. As a functional imaging modality, PET imaging has become a vital tool in oncology for initial staging, detection of regional and distant metastases, identification of tumor recurrences, and assessment of therapeutic response [7,8,9,10]. Radiomics is a machine learning-based technology for quantitative image analysis. As a machine learning-driven quantitative image analysis technique, radiomics has emerged as an active area of research for its ability to deliver a comprehensive and detailed representation of the radiographic phenotype of a 3D target volume [11,12,13,14,15,16,17]. This capability has resulted in a growing number of applications for radiomics in risk assessment and predicting treatment response in cancer management [18,19,20,21].

PET radiomics has achieved promising initial success in managing lung cancer risk; nevertheless, challenges remain concerning the standardization of methods and the generalization of models [22,23,24]. Since radiomics analysis heavily relies on the volume of interest, variations in delineation (segmentation) can significantly affect the robustness of the extracted features, thereby undermining the generalizability of the generated predictive models. Furthermore, several limitations present significant challenges in achieving consistent and reproducible manual segmentation (delineation) across different observers on PET imaging. These limitations include PET images often appearing blurred, and variations in display settings can impact volume perception. While automatic, or semi-automatic, segmentation is less prone to these limitations compared to manual segmentation, it is still subject to variations caused by intrinsic difference in image quality among different image scanners and reconstruction algorithms.

The goal of this study was to identify key radiomics features that are stable against these variabilities and enhance risk stratification for predicting survival benefits in stage IVB non-small cell lung cancer using these features.

## 2. Materials and Methods

### 2.1. Patient Characteristics

The Institutional Review Board at our institution approved this retrospective study (IRB 722-19-EP) and waived the requirement of obtaining informed consent from the subjects. A total of 99 stage IVB NSCLC patients were retrospectively selected for this study, 40 males and 59 females, with a median age of 67, ranging from 30 to 88 years, at the time of diagnosis. Histological diagnosis was determined by thoracic pathologists and was retrieved and recorded from the Electronic Medical Record (EMR). Other clinical data including gender, race, history of tobacco use, chronic obstructive pulmonary disease (COPD), and coronary artery disease (CAD) were collected. Additionally, biomedical parameters such as BMI at diagnosis, platelet count, and neutrophil count at the onset of treatment were collected. The first-line treatment regimens were also recorded. The patient characteristics are summarized in Table 1.

### 2.2. Volume of Interest Segmentation

**Figure 1:**
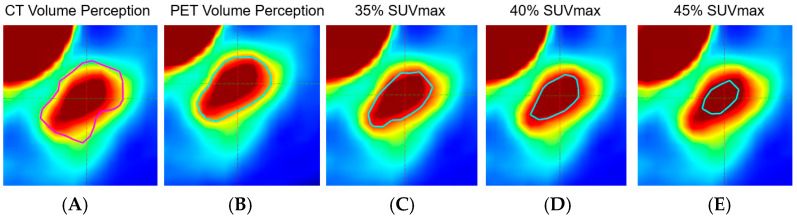
Contour variation based on (**A**) volume perception on CT; (**B**) volume perception on PET; (**C**) 35% of SUV_max_; (**D**) 40% of SUV_max_; (**E**) 45% of SUV_max_. The color scale in the PET scans represent levels of metabolic activity: red and yellow indicate high activity, and green and light blue show moderate or low activity.

The primary lung lesion of each patient was delineated on PET/CT scans prior to diagnosis through consensus between an attending radiation oncologist and an attending medical oncologist using the Varian Eclipse treatment planning system (Varian Medical Systems, Palo Alto, CA, USA). To account for the variations caused by factors such as scanner type, reconstruction, and image noise of the PET images, we generated three delineation variations using fixed threshold values of Maximum Standardized Uptake Value (35% SUV_max_, 40% SUV_max_, and 45% SUV_max_), inspired by a previous study [23] with adjustments. Additionally, to address the variations arising from the possibility that the volume perception on PET images (PET hotspot area) may not perfectly align with the volume perception on CT images (tissue level density in lung), we also delineated the primary lung lesion based on the CT volume perception, using the preset lung window/level in Eclipse, on the PET images. Figure 1 showed all five contour variations we generated. All the segmentations were delineated on the images with their original in-plane resolutions and slice thicknesses. The images with associated segmentations were stored and exported via the DICOM format for processing and analysis.

### 2.3. Feature Extraction

Open-source software PyRadiomics, version 3.0.1 [25] was used to extract radiomics features from the set of PET images. DICOM images and target delineation were converted to NRRD format using a batch process in 3D slicer software [26]. Nine hundred and twenty-four (924) radiomics features were extracted from the primary lung lesion. The radiomics features included first-order statistics, 3D shape-based features, a Gray-Level Co-Occurrence Matrix (GLCM), Gray-Level Run Length Matrix (GLRLM), Gray-Level Size Zone Matrix (GLSZM), Neighboring Gray-Tone Difference Matrix (NGTDM), and Gray-Level Dependence Matrix (GLDM) from the original images, images derived from Laplacian of Gaussian (LoG) filters, and eight derived images from wavelet decompositions [16].

### 2.4. Clinical Endpoints

In this study, we chose to assess the role of radiomics features in modeling patients’ overall survival and 1-year survival. The overall survival was calculated from the date of diagnosis to the date of death. For 1-year survival, we also grouped the patients into alive or deceased categories at 1 year for a classification model.

### 2.5. Uncertainty Analysis of Radiomics Features

In radiomics research, feature selection is a crucial step in the workflow due to the typically large number of available features compared to the limited number of cases in the patient cohort. Failure to perform feature selection may lead to overfitting [27], where the model may not generalize well to independent datasets and demonstrate accurate predictive power. To assess the stability and reproducibility of the extracted features, we introduced two perturbations in the image preprocessing and feature extraction process, focusing on variations in contour and extraction parameters (bin width and with/without resampling). The goal is to ensure that extracted features are robust against these variabilities such that the model can effectively adapt to new datasets. The robustness of features with respect to these perturbations was assessed using the Intraclass Correlation Coefficient (ICC) [28], where unstable features are removed from the final dataset for performing classification and predictive analysis.

### 2.6. Contour Variation

Delineation of tumors on PET images is challenging due to the intrinsic limitations of PET images, such as low resolution and significant noise levels. Manual contouring is commonly used in the clinical setting, but it often results in variability because factors such as PET images appearing blurred and display settings, such as window level and width, can affect volume perception. Automatic or semi-automatic segmentation methods are advantageous in improving efficiency by reducing delineation time and inter-operator variability. However, the reproducibility of automatic segmentation can still be undermined by variabilities in image scanners, reconstruction algorithms, and image noise, among others. As mentioned earlier, in addition to the delineation based on volume perception on the PET images, we created four variations: one based on volume perception on the CT images using a preset lung window level (later transferred to the PET images), and three using fixed threshold values [29] of SUV_max_ (35%, 40%, and 45%).

### 2.7. Bin Width and Resampling

Two important feature extraction parameters include image voxel size and the extraction bin width. We aimed to investigate how different bin widths and resampling techniques (with or without resampling) impact the stability of the extracted features. Three different gray-level discretization were chosen for voxel intensity values, 5, 25, and 75, and were applied with the original resolution and a resampled resolution of 1.0 mm × 1.0 mm × 1.0 mm. Original planar resolutions ranged from 2.7 mm × 2.7 mm to 5.5 mm × 5.5 mm, and original slice width ranged from 2 mm to 5 mm. The combination of the bin width and resampling allowed for a total of six different parameter sets to be explored for feature robustness. ICC > 0.75 was again the threshold for the determination of the robust features using different feature extraction parameters.

### 2.8. Feature Stability Evaluation

The Intraclass Correlation Coefficient (ICC) is the statistical measure used to quantitatively assess the robustness of radiomics features against various perturbations. In this study, the PET features were extracted under different perturbations to the dataset (bin width and contour variation) and evaluated for robustness using the ICC. Specifically, the ICC(2,1) was selected to assess the absolute agreement with the 2-way random effects model since this 2-way random effects model was the appropriate model to generalize our reliability results [28,30], calculated as follows:

$$\mathrm{ICC}(2,1)=\frac{MS_{R}-MS_{E}}{MS_{R}+\left(K-1\right)MS_{E}+\frac{K}{N}\left(MS_{C}-MS_{E}\right)}$$

where MS_R_, MSc, MS_E_ is the statistical mean square for R = rows, C = columns, and E = error, respectively, for the radiomics feature dataset.

### 2.9. Train/Test Data Split

**Figure 2:**
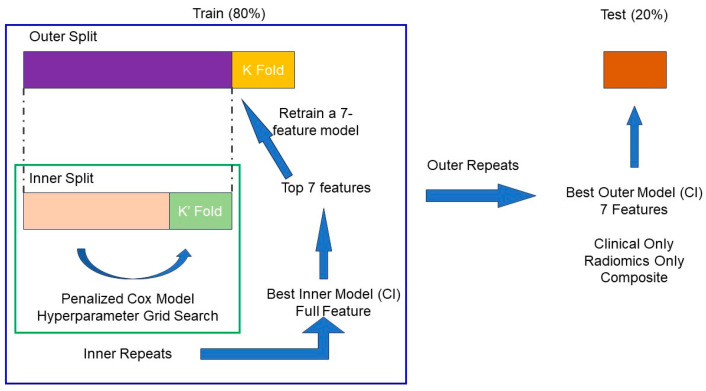
In-house workflow for survival analysis.

As shown in Figure 2, the patients were randomly split into a training set (80%) and a test set (20%), while ensuring the classification event was balanced and the categorical variables were stratified. We utilized the same train/test split for our clinical, radiomics and composite models for both survival analysis and classification prediction. All continuous variables were normalized using min–max normalization, while categorical variables were encoded using one-hot encoding, a method that converts categories into binary vectors, allowing the model to process categorical data more effectively.

### 2.10. Survival Analysis Workflow Utilizing a Penalized Cox Model

As shown in Figure 2, we designed a survival analysis workflow that utilized a nested cross-validation approach to optimize a Cox Proportional Hazards Model with elastic net regularization (penalized Cox model [31]) for survival analysis. Briefly, our workflow repeatedly split the training data (80%) into outer training set (purple) and outer test set (orange) using 5-fold cross-validation. Within each outer training set (purple), it performed multiple inner cross-validation repetitions (green box), exploring various ‘l1_ratio’, ‘alpha_min_ratio’, and ‘alpha’ combinations using a grid search approach to identify the best hyperparameters. Then, the features with non-zero coefficients were ranked based on the absolute value of their coefficients and stored for each inner repetition. The 7 best features, which were selected based on frequency during the inner repetitions, were utilized to create a penalized Cox model in the outer training set and evaluated by the outer test set. The best-performing 7-feature model was determined by its concordance index (CI) from the outer loop. Then, this best performing 7-feature model was evaluated using the independent test set (20% of the total patients) using the concordance index (CI). Three models were trained using the same training set (79 patients), with one model fed clinical features alone, another with radiomics features alone, and the third with a combination of both clinical and radiomics features, as illustrated in this workflow (Figure 2).

### 2.11. One-Year Survival Classification Predictive Modeling

In addition to the survival models mentioned above, three classification models were trained utilizing the Balanced Random Forest (BRF) framework on the same training set (79 patients), as mentioned in the Train/Test Data Split Section, to differentiate the alive and deceased cases at 1 year. These models utilized clinical features, radiomics features, and a combination of both clinical and radiomics features (composite model), respectively. Each model utilized the same 7 features as identified in the survival analysis, as mentioned earlier. We utilized a grid search approach to optimize the hyperparameters of the BRF classifier using our in-house classification workflow [32,33]. We explored a parameter grid consisting of ‘max_depth’, ‘n_estimators’, ‘min_samples_split’, ‘min_samples_leaf’, ‘max_features’, ‘bootstrap’, and ‘criterion’ with specific value ranges. GridSearchCV was utilized to perform the grid search with 5-fold cross-validation on the training set. The BRF classifier model was then constructed using the best hyperparameters identified and tested on the test dataset for performance evaluation. The performance of the models was quantified by the area under the receiver operating characteristic curve (AUC), accuracy, sensitivity, and specificity.

### 2.12. Implementation and Programming Environment

Our study was implemented using in-house programming within the Python environment, allowing for flexibility and customization in data processing, model development, and analysis. Python was chosen for its extensive libraries suited to scientific computing, data processing, and machine learning. The following Python packages were used in this study: “NumPy”, “Pandas”, “Scikit-learn”, “Matplotlib”, “Seaborn”, “Pingouin”, “Imbalanced-learn”, “SimpleITK”, “Pydicom”, and “scikit-survival”. This Python-based setup enabled a streamlined and reproducible workflow, facilitating the integration of diverse methodologies required for the study.

## 3. Results

### 3.1. Feature Extraction and Uncertainty Analysis

**Figure 3:**
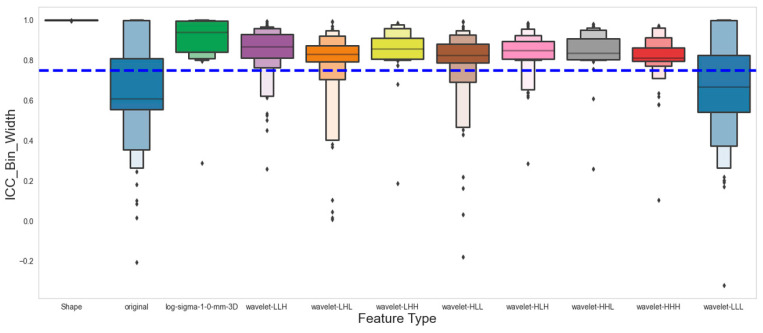
Intraclass Correlation Coefficient of PET radiomics features against extraction parameters as shown in the Boxen Plot. The blue dashed line marks where the ICC is 0.75, and the dots represent individual data points that are often outliers.

**Figure 4:**
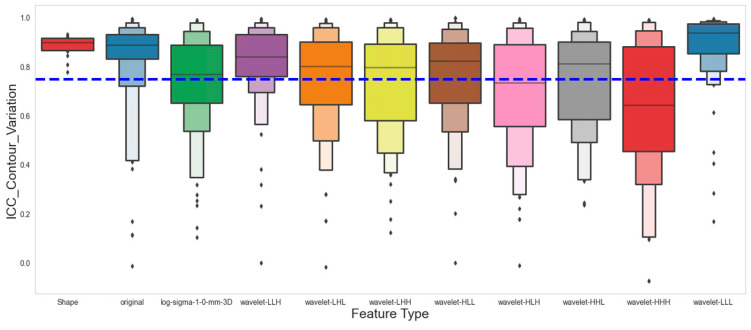
Intraclass Correlation Coefficient of PET radiomics features against contour variation as shown in the Boxen Plot. The blue dashed line marks where the ICC is 0.75, and the dots represent individual data points that are often outliers.

A total of 924 PET radiomics features were extracted for each volume of interest on each patient. To account for variability in extraction parameters, we used six different sets of extraction criteria, consisting of three bin widths (5, 25, 75) and images with or without resampling. Figure 3 shows the Intraclass Correlation Coefficient (ICC) of all the features, grouped by feature class. Our analysis has revealed that 80.6% (745 out of 924) of the extracted features are robust against various extraction parameters. Furthermore, by comparing feature consistency across the five contour variations, we found that 63.9% of the features (590 out of 924) remained consistent across contour variations using both manual and threshold-based automatic segmentation (Figure 4).

### 3.2. Feature Selection

**Figure 5:**
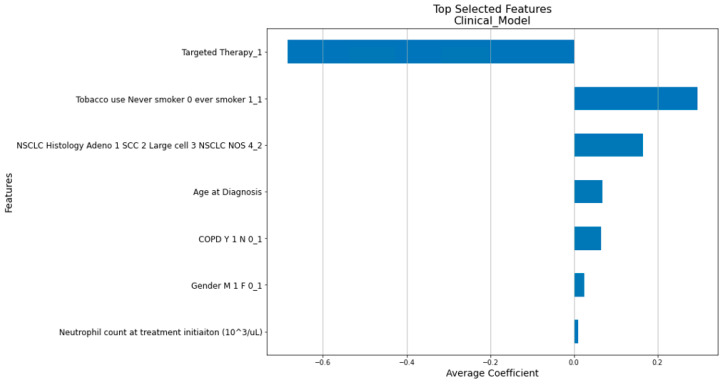
The 7 selected clinical factors with their coefficients.

**Figure 6:**
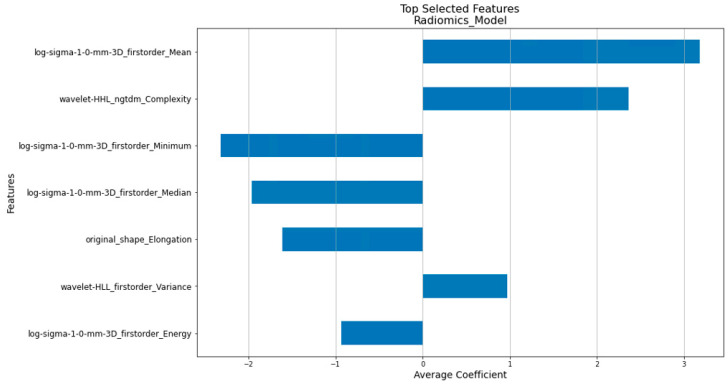
The 7 selected PET radiomics factors with their coefficients.

**Figure 7:**
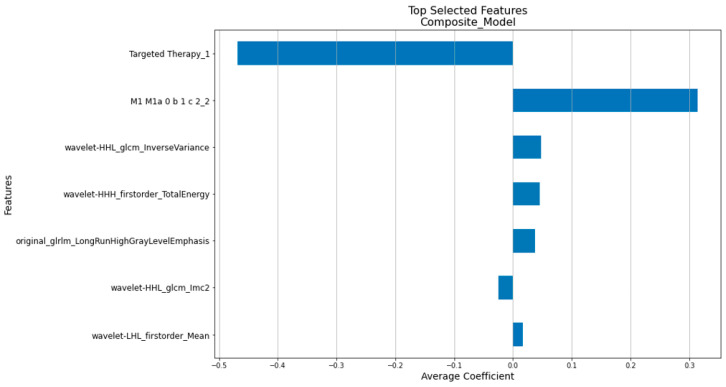
The 7 selected composite factors with their coefficients.

Compared to the traditional Cox Proportional Hazards Model, the penalized Cox model [31] incorporates L1 and/or L2 penalties in the Cox Proportional Hazards Model. This enhancement allows it to perform feature selection and survival analysis simultaneously, making it more suitable for handling large and correlated datasets. As shown in Figure 2, the features of the clinical, radiomics, and composite models were determined by our in-house workflow involving the use of the penalized Cox model. Figure 5, Figure 6 and Figure 7 show the top features selected by our workflow hosting the penalized Cox model for the clinical, radiomics, and the composite model.

### 3.3. Survival Model Performance

**Figure 8:**
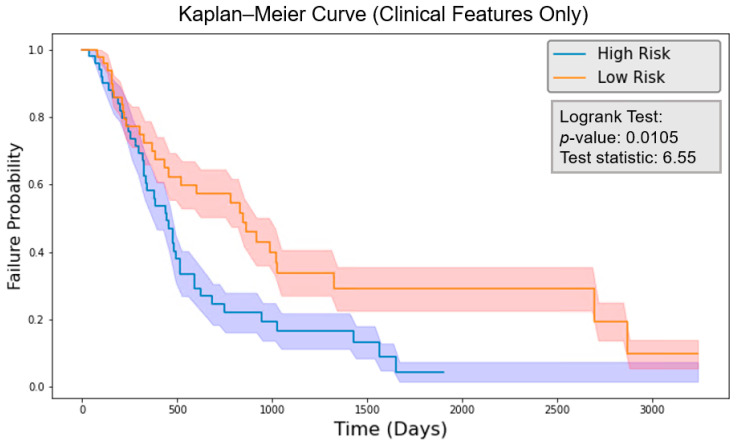
Kaplan–Meier curve for the model with clinical features only. The shadowed region indicates one standard deviation above and below the mean.

**Figure 9:**
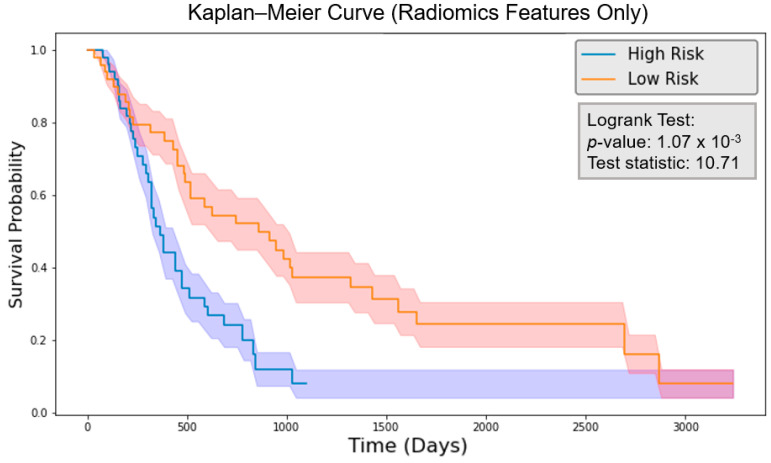
Kaplan–Meier curve for the model with PET radiomics features only. The shadowed region indicates one standard deviation above and below the mean.

**Figure 10:**
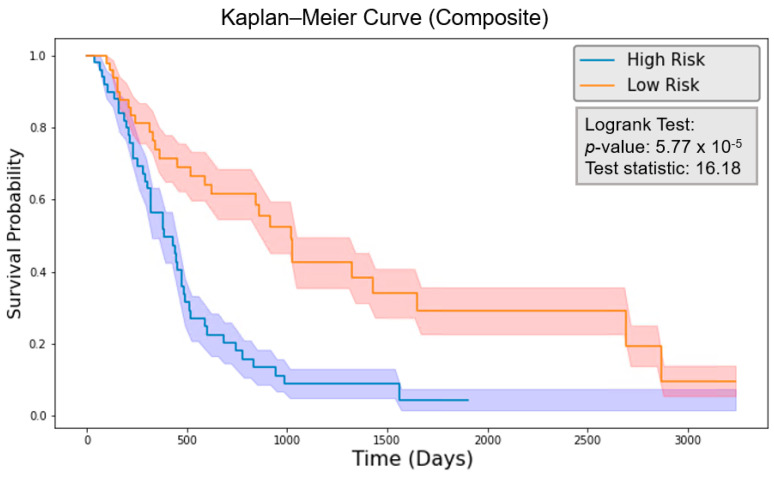
Kaplan–Meier curve for the model with composite features. The shadowed region indicates one standard deviation above and below the mean.

The risk score was calculated by the final 7-feature model with the feature values of each patient. The patients were grouped by comparing their risk scores to the mean risk score of all test patients (20 patients). Those with risk scores above the mean were classified as the high-risk group, while those with scores below the mean were classified as the low-risk group for each model. The logrank test was performed, and the *p*-value was calculated. As shown in Figure 8, the clinical model obtained a logrank test result of 6.55 with a *p*-value of 0.0105. Figure 9 indicates that the radiomics model reached a logrank test result of 10.71 with a *p*-value of $1.07\times 10^{-3}$. Meanwhile, Figure 10 shows that the composite model achieved a logrank test result of 16.18 with a *p*-value of $5.77\times 10^{-5}$. The three models achieved concordance index (CI) values of 0.604, 0.623, and 0.662 when applied to the same test set (Table 2).

### 3.4. Classification Model Performance

**Figure 11:**
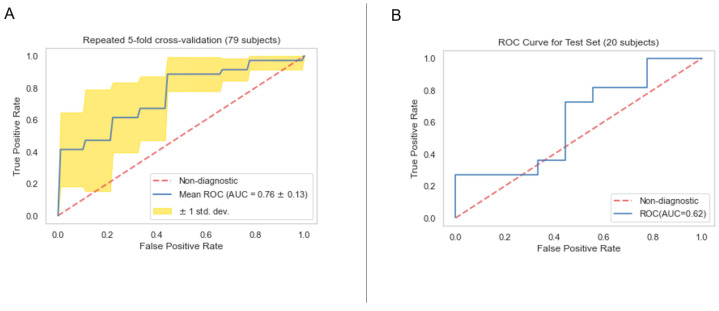
ROC curves for the training (**A**) and test set (**B**) of the clinical classification model.

**Figure 12:**
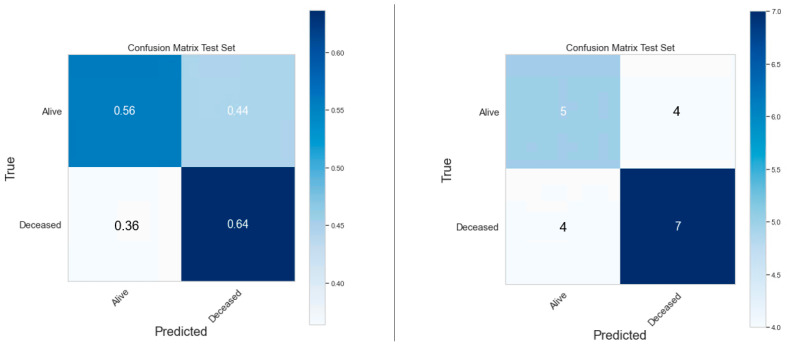
Confusion matrix of the clinical classification model.

**Figure 13:**
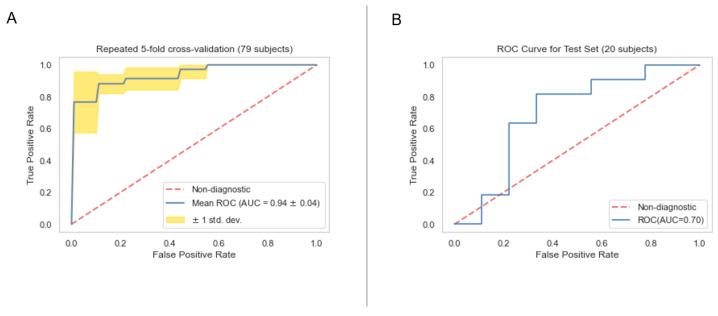
ROC curves for the training (**A**) and test set (**B**) of the radiomics classification model.

**Figure 14:**
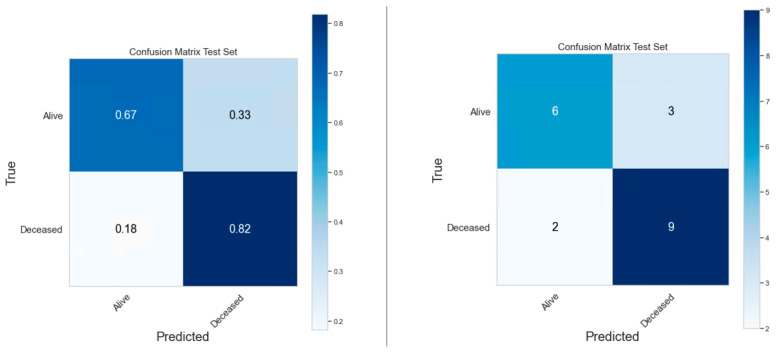
Confusion matrix of the radiomics classification model.

**Figure 15:**
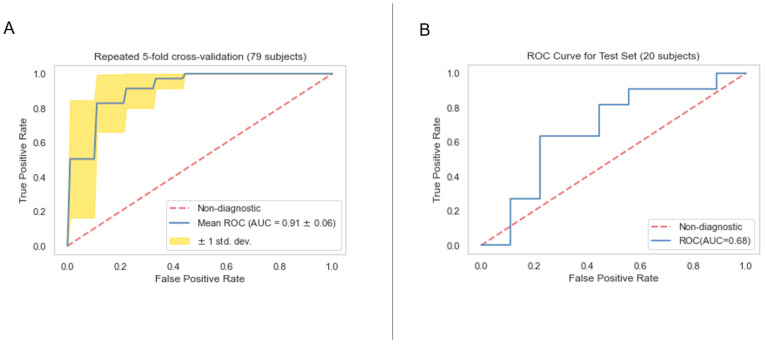
ROC curves for the training (**A**) and test set (**B**) of the composite classification model.

**Figure 16:**
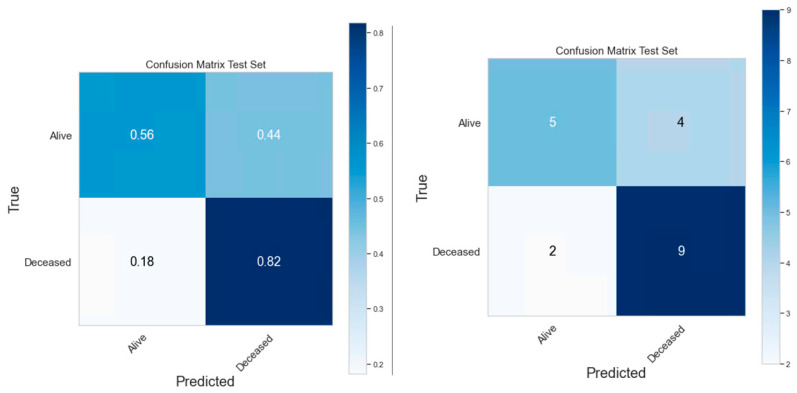
Confusion Matrix of the composite classification model.

The performances of clinical model, PET radiomics model, and the composite model are summarized in Figure 11–Figure 12, Figure 13–Figure 14 and Figure 15–Figure 16, respectively. As shown in Figure 11A, Figure 13A and Figure 15A, repeated 5-fold cross-validation on the training dataset (79 patients) demonstrated that the clinical, radiomics, and composite models achieved average area under receiver operating characteristic curve (AUC) values of 0.76 (95% CI: 0.63–0.89), 0.94 (95% CI: 0.9–0.98), and 0.91 (95% CI: 0.85–0.97). Upon evaluation with the same independent test dataset of 20 patients, each model achieved AUC values of 0.62, 0.7, and 0.68 (Figure 11B, Figure 13B and Figure 15B). The prediction accuracies of the three models were 0.6, 0.75, and 0.7 (Table 2). Figure 12 shows that the clinical model achieved a sensitivity of 0.64 and a specificity of 0.56. The PET radiomics model demonstrated a sensitivity of 0.82 and a specificity of 0.67 (Figure 14). Meanwhile, Figure 16 illustrates that the composite model achieved a sensitivity of 0.82 and a specificity of 0.56. The prediction accuracy of each model is 0.5, 0.7, and 0.65, respectively (Table 2).

## 4. Discussion

Despite advances in treatment, stage IV non-small cell lung cancer (NSCLC) still has low overall cure and survival rates, with major management challenges due to disease heterogeneity, resistance to standard chemotherapies, and the limited success of targeted therapies and immunotherapies [4,5,6]. PET imaging, with its capacity to uncover metabolic signatures of the disease, has become an important standard in cancer staging, treatment planning, and evaluating treatment response. PET radiomics has shown promising potential in stratifying lung cancer risk; however, challenges still exist in standardizing methods, such as determining the best approach between manual and automatic segmentation and generalizing radiomics-based models.

In the present study, we investigated the impact of contour variation, introduced by manual and semi-automatic segmentation, on the stability of the PET radiomics features. Specifically, we delineated the primary lung lesions using three threshold values of the SUV_max_ (35%, 40%, and 45%), and we also delineated the lesions on PET images based on volume perception on both PET and CT images. Then, we calculated the ICC to evaluate feature stability of the 924 features. Our results showed that 63.9% of the features are stable against contour variation The rationale for generating these variations is that volume perception-based manual segmentation and fixed threshold-based automatic segmentation are the most commonly available methods in clinical settings compared to other methods [23] that have been investigated for their impact on feature stability. Given their widespread availability, the contouring methods we examined are likely the most accessible for new data collection for potential external validation and continuous assessment of our predictive models.

We also examined the impact of the extraction parameters (bin width and with/without resampling) on feature robustness, and we showed that 80.6% of the features are robust against variations in the extraction parameters. This percentage is notably higher than the stability of CT radiomics features against extraction parameters observed in our previous results [32]. This is likely a result of PET images typically having low resolution and relatively higher noise level compared to CT.

With the common 367 final features that are stable against both perturbations, we established two classes of models, a survival prognostic model and a 1-year survival classification model. Within each class, we developed three models using clinical features only, radiomics features only, and the combined set of features. Our results showed that PET radiomics features, either alone or combined with clinical features, can successfully stratify patient survival risk. The radiomics model and composite model achieved a logrank test of 10.71 and 16.18 with *p*-value less than 0.05 (Figure 9 and Figure 10). Their concordance index (CI) was 0.623 and 0.662 when applied to the independent test set. Additionally, the classification model showed that PET radiomics features alone or in combination with clinical factors can achieve better prediction accuracy compared to clinical factors alone, with an AUC of 0.7 and 0.68 (Figure 13 and Figure 15) and a predicting accuracy of 0.75 and 0.7, respectively (Table 2).

Our study has a few limitations. We have a relatively small sample size from a single institution, and our conclusions necessitate cautious interpretation and require validation using external datasets. The observed discrepancy in AUC values between the training set and the test set suggests that the sample size may be insufficient for robust model evaluation. Future investigations following our proof-of-concept study are still warranted. Our model needs to be validated, and possibly evolved, with external datasets. We also plan to perform independent tests using newer patients.

## 5. Conclusions

Robust PET radiomics analysis successfully facilitated the stratification of patient risk for survival outcomes and predicted 1-year survival in stage IVB Non-Small Cell Lung Cancer.

## Figures and Tables

**Table 1:** Patient characteristics.

Patient Characteristics	
**Gender**	
Female	59
Male	40
**Age**	
Median	67
Range	30–88
**Smoking History**	
Ever Smoked	76
Never Smoked	23
**Stage**	
M1a	22
M1b	13
M1c	64
**First-Line Treatment Regimen**	
Chemotherapy	56
Targeted Therapy	30
Immunotherapy	9
Chemo-Immunotherapy	4
**Histology**	
Adenocarcinoma	79
Squamous Cell Carcinoma	15
Large Cell	3
NSCLC NOS	2

**Table 2:** Concordance index and prediction accuracy on the test set.

Model	Concordance Index (Survival Model)	1-Year Survival Prediction Accuracy (Classification Model)
**Clinical**	0.604	0.6
**Radiomics**	0.623	0.75
**Composite**	0.662	0.7

## Data Availability

The datasets presented in this article may not be readily available due to privacy and ethical restrictions. Requests to access the datasets should be directed to the corresponding author.
